# Simulation-based evaluation of SAR and flip angle homogeneity for five transmit head arrays at 14 T

**DOI:** 10.1007/s10334-023-01067-1

**Published:** 2023-03-31

**Authors:** Seb D. Harrevelt, Thomas H. M. Roos, Dennis W. J. Klomp, Bart R. Steensma, Alexander J. E. Raaijmakers

**Affiliations:** 1 Department of Biomedical Engineering, Eindhoven University of Technology, Eindhoven, The Netherlands; 2 Department of Radiology, University Medical Center Utrecht, Utrecht, The Netherlands

**Keywords:** Simulation study, 14T, SAR assesment, Coil designs, Flip angle homogeneity

## Abstract

**Introduction:**

Various research sites are pursuing 14 T MRI systems. However, both local SAR and RF transmit field inhomogeneity will increase. The aim of this simulation study is to investigate the trade-offs between peak local SAR and flip angle uniformity for five transmit coil array designs at 14 T in comparison to 7 T.

**Methods:**

Investigated coil array designs are: 8 dipole antennas (8D), 16 dipole antennas (16D), 8 loop coils (8D), 16 loop coils (16L), 8 dipoles/8 loop coils (8D8L) and for reference 8 dipoles at 7 T. Both RF shimming and *k*_*T*_-points were investigated by plotting L-curves of peak SAR levels vs flip angle homogeneity.

**Results:**

For RF shimming, the 16L array performs best. For *k*_*T*_-points, superior flip angle homogeneity is achieved at the expense of more power deposition, and the dipole arrays outperform the loop coil arrays.

**Discussion and conclusion:**

For most arrays and regular imaging, the constraint on head SAR is reached before constraints on peak local SAR are violated. Furthermore, the different drive vectors in *k*_*T*_-points alleviate strong peaks in local SAR. Flip angle inhomogeneity can be alleviated by *k*_*T*_-points at the expense of larger power deposition. For *k*_*T*_-points, the dipole arrays seem to outperform loop coil arrays.

**Supplementary Information:**

The online version contains supplementary material available at 10.1007/s10334-023-01067-1.

## Introduction

Increasing the B_0_ field strength remains a focal point of interest within MRI research due to expected gains in SNR, CNR, and spectral dispersion. In the last 5 years, the first imaging results of 10.5 T and 11.7 T human systems have been published and multiple other research sites are currently bringing 11.7 T MRI systems into operation [1–5]. More recently, various research sites across the world are planning to install 14 T MRI [6] systems with improved resolution of fMRI and spectroscopic imaging MRSI to provide a better understanding of human brain function.

Previous work at ultra-high field strength has uncovered the challenges that come with increasing B_0_ field strength. Due to the shortening RF wavelength, both local SAR and transmit field inhomogeneity increase with field strength and have a negative impact on image quality and scan efficiency [7, 8]. At 7 T–9.4 T, acquisition of uniform image contrasts throughout the brain is feasible with improved SNR compared to 3–4 T [9, 10]. However, as soon as larger objects such as the body are studied at these field strengths, efficient acquisition of uniform images becomes very challenging [8]. A large amount of research over the past years has shown that improvements in the RF coil design and parallel transmission can be used to significantly alleviate problems with image contrast uniformity and local SAR at 7 T–10.5 T [11–21].

When further increasing the B_0_ field strength to 14 T, it becomes questionable if the short RF wavelength will still allow for efficient imaging of the brain. At 14 T, the RF wavelength in white matter is ~ 6 cm which is significantly smaller than the dimensions of the head and which will likely cause destructive interference and standing wave patterns in the brain. To determine the feasibility of human brain imaging at 14 T, the purpose of this study is two-fold. We want to 1. Investigate trade-offs between local SAR and B_1_^+^ uniformity in the human brain at 14 T for various RF shimming and parallel transmit approaches as compared to 7 T 2. Investigate the impact of coil design on local SAR and flip angle uniformity at 14 T.

Although no 14 T MRI system yet exists for human applications, the expected imaging performance and short-wavelength penalties can be well investigated using numerical simulations. As an example, the flip angle homogeneity and SAR levels for various body imaging targets of the 10.5 T system currently operational at CMRR, Minneapolis, has been investigated beforehand [22]. This study thoroughly investigated the flip angle homogeneity and peak SAR levels that were to be expected once the system would be available for human scanning. The authors investigated the performance for RF shimming and parallel transmit (spokes pulses) and used L-curves to depict the trade-off between flip angle homogeneity and peak local SAR (further referred to as peak SAR).

Similarly, another simulation study has been performed to compare SNR, SAR and flip angle uniformity in the brain at 1.5–14 T [23]. This study thoroughly demonstrated the expected gains in signal-to-noise ratio for increasing field strengths for realistic pulse sequences and demonstrated that is was feasible to achieve uniform flip angle distributions in the brain at 14 T. However, the expected penalties in terms of SAR and B1 inhomogeneity represent an upper limit as this study only included one single coil array design, which was not optimized for the task at hand. Also, it did not study the trade-off between B1 field uniformity and peak SAR. Depending on the RF pulse design settings, users can choose either better uniformity or lower SAR levels. On top of this, the use of parallel transmission pulse designs (e.g. *k*_*T*_-points) allows for further optimization of flip angle homogeneity which was only sparsely investigated (i.e. without evaluating peak SAR for these pulses). The aim of the current study is to investigate the trade-offs between peak SAR, B_1_^+^ and flip angle uniformity at 14 T in comparison to 7 T using numerical simulations. As findings will depend heavily on the chosen coil array design, the investigation is performed for a range of potential coil arrays for brain imaging at 14 T. L-curves will show for each array the trade-off between peak SAR and flip angle homogeneity using either RF shimming or 5-point *k*_*T*_-points pulse. Next to the expected RF transmit performance at 14 T, the study also provides initial directions in RF coil array design for 14 T brain imaging and provides a more complete picture of local SAR behavior at 14 T compared to 7 T.

## Methods

### EM simulations setup

**Fig. 1:**
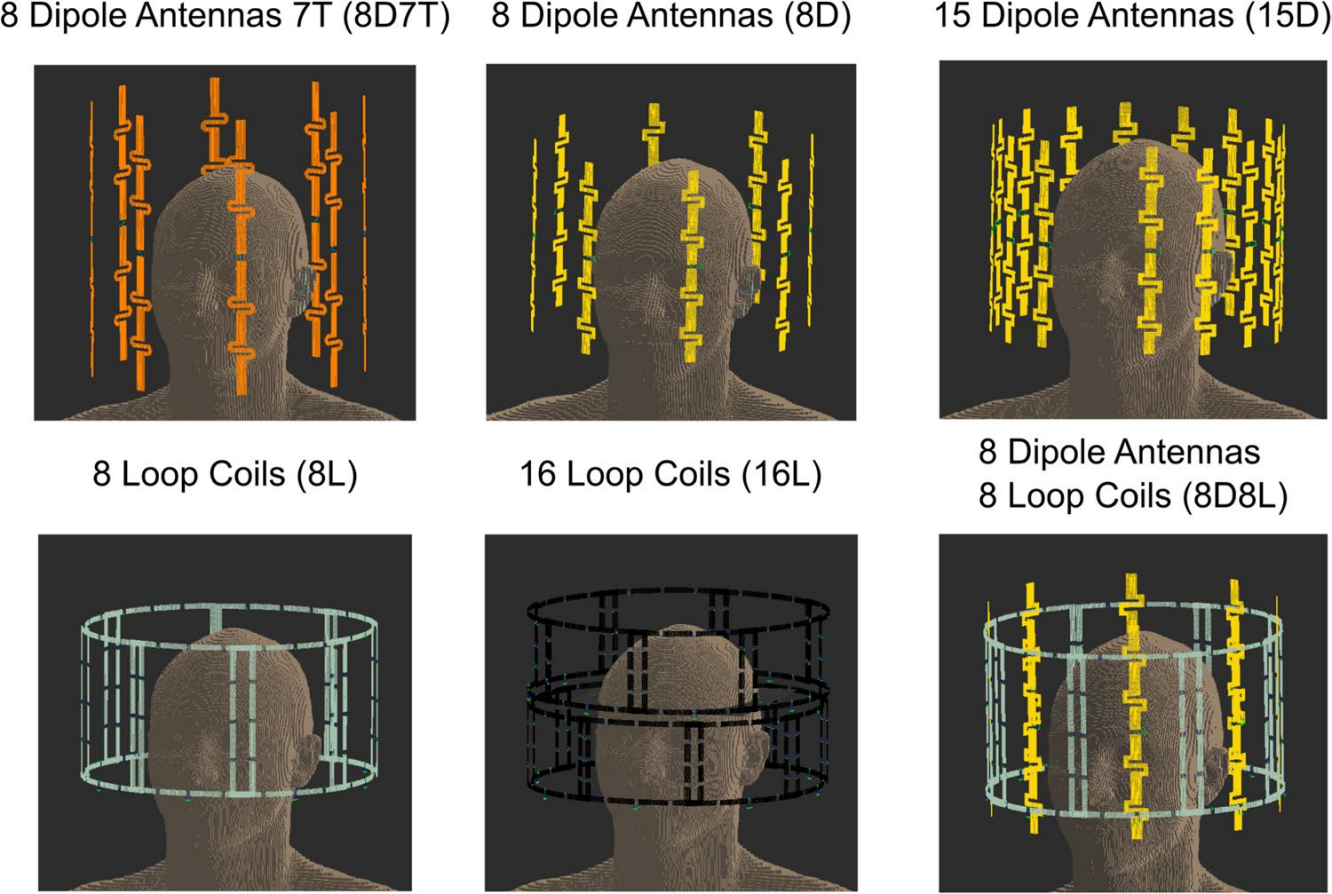
Visualization of the proposed coil array designs used in Sim4Life

Finite difference time domain (FDTD) simulations were performed in Sim4Life (Zurich Medtech, Zurich, Switzerland). Five different transmit array configurations were simulated at 596 MHz (14 T), using the following transmit elements: 8 dipole antennas (8D), 15 dipole antennas (15D), 8 loop coils (8D), 16 loop coils (16L), 8 dipoles and 8 loop coils (8D8L). An array of 8 fractionated dipoles was also simulated at 298 MHz (7 T) as reference. Figure 1 shows a visualization of the proposed coil designs. These simulations were performed on the brain of human model Duke of the virtual family [24].

All antennas were placed on a ring with a diameter of at least 300 mm. In case of adjacent overlapping elements, one of the elements was placed 5 mm further away from isocenter. For 14 T, all array were kept within a total feet-head length between 16 cm (8L) and 20 cm (all other arrays), whereas at 7 T an optimal dipole length of 30 cm was used [16].

The dipole antennas in configuration I, II and V had a length of 20 cm, according to optimal results from a previous 14 T simulation study at 14 T [25]. The dipole antennas were matched with a parallel capacitor (2.45 pF) and two parallel inductors (14 nH). The dipole antennas at 7 T had an optimal length of 30 cm [16] and were matched with a parallel inductor of 40 nH and two series inductors of 25 nH.

The large loop coils of array 8L and 8D8L had a height of 16 cm and a width of 14 cm (optimal for low SAR at 7 T [26]) and were tuned with 1.19 pF capacitors (12 per loop). The small loops had a height of 87.5 mm and a width of 14 cm. Loops were separated in the feet head direction by a gap of 5 mm (total array length 20 cm). The small loops were tuned with 12 × 1.85 pF capacitors. To match the loops, the port reference impedance was set equal to the real part of the coil impedance. All loops were overlapped by 1 cm, which resulted in minimum nearest-neighbor coupling values for an array diameter of 300 mm at 14 T.

All the coils in this work include a 62 cm diameter RF shield (gradient shield) and are placed on a 30 cm diameter ring.

A grid size of 1–1.5 mm^3^ was used to voxelize the antennas, lumped elements and ports. The head and shoulders were surrounded by a bounding box which was voxelized at an isotropic grid size of 2.5 mm^3^ to ensure that all body parts where high local SAR could occur were properly included in the model. Total grid size was between 15 and 31 million cells depending on the array complexity. All simulations were performed on a GPU (Nvidia Titan RTX, Nvidia, Santa Claura, USA) and took up to 1 h per port.

Electric and magnetic fields were exported from Sim4Life into Matlab (Mathworks, Natick, USA). After calculating Q-matrices in Matlab, a custom 10 g averaging script [27] was used to calculate 10 g-averaged Q-matrices, which were then compressed into virtual observation points [28] (VOPs)) which allows calculation of peak SAR The compression caused a SAR overestimation of 2.5% for all investigated simulations.

### Numerical analysis of EM simulations

**Fig. 2:**
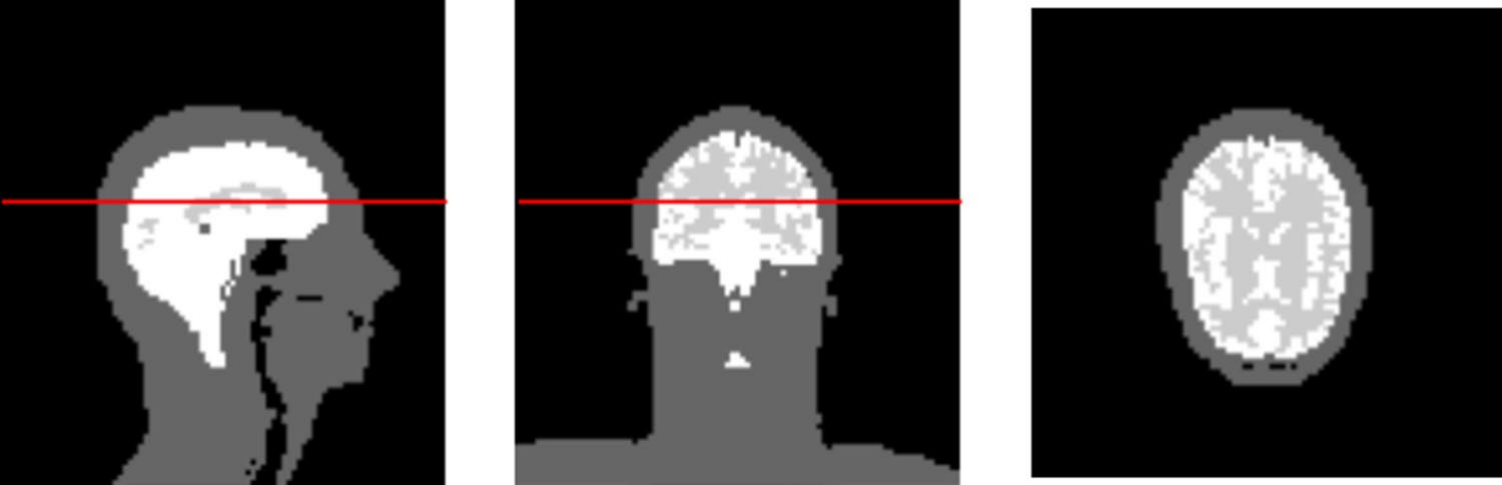
Three slices of the brain mask used for optimization. The region covered by the white area is the used mask. To aid in the orientation, we added the gray matter of the brain imaged by the light gray area inside this mask. The red line indicates the middle slice that is used for visualization

The simulated B_1_^+^ distributions and VOPs were used to find the optimal performance of each coil design. For this, either amplitude/phase shimming or the *k*_*T*_-points method [29] was used to design optimal non-selective pulses. Both methods were optimized and evaluated on the same 3D brain mask shown in Fig. 2.

#### RF shimming

For RF shimming, we choose to optimize the following cost function, which is a tradeoff between flip angle uniformity (normalized-root-mean-square error, NRSME) and forward power.

1 $${\mathrm{min}}_{x}|||Ax|-b||+\lambda ||x|{|}_{2}^{2}$$

The regularization parameter λ was varied between zero and 1/10 with 25 equidistant increments, this range was found empirically to reflect the best L-curve.

This minimization problem was solved by Conjugate Gradients [30] using random uniform shim coefficients as initial solution. To prevent the convergence to a single local minimum we repeated this for 25 random initial solutions.

With these solutions we numerically calculated the peak SAR and NMRSE which form an L-curved shape when plotted on a 2D grid. From this, the optimal shim coefficients were visually selected near the strongest curvature since the L-curves suffered from irregularities that complicated automatic selection.

In addition to the optimized shim coefficients we also simulated 10 000 random shim settings. These were chosen from a random uniform distribution with an amplitude ranging between 0 and 4, and a phase variation between 0 and 2π. These were used to gain insight into the general response in SAR between 7 and 14 T.

#### *k*_*T*_-points

Non-selective *k*_*T*_-points pulses consisting of multiple block subpulses, with gradient blips in between were optimized using the interleaved greedy and local algorithm [31]. The optimization of the complex shims for each subpulse and their *k*_*T*_-points locations targeted a flip angle of 40°, with an initial phase distribution equal to the phase of the summed B_1_ distributions. The first of set minimization problems, with a increasing number of *k*_*T*_-points subpulses, was regularized with forward power, where the regularization parameter had an initial value of 20 and was adjusted during the optimization process.

In subsequent optimizations, we optimized *k*_*T*_-points pulses containing a fixed number of 5 sub pulses, with forward power as fixed regularization. In this setting we varied the value of the regularization parameter in 20 exponential steps, to also demonstrate the trade-off between flip angle homogeneity and peak SAR.

#### Normalization flip angle

To compare the results of merely RF shimming versus *k*_*T*_-points, we used a linear scaling factor such that an average flip angle of 40° was reached inside the mask and a pulse of length 0.76 ms was used. This pulse length corresponds to the pulse length from the *k*_*T*_-points simulations. Choosing these settings corresponds to an average B_1_^+^ level of 3.4 µT.

#### Normalization to head SAR

We used the power deposition matrix to normalize the SAR distributions to 3.2 W/kg head SAR. This matrix is calculated by the following equation

$${\phi }_{ij}=\frac{1}{2}\int \sigma {{\varvec{E}}}_{i}\cdot {\overline{{\varvec{E}}} }_{j}d{\varvec{V}}, i,j=1, 2, \dots , {n}_{\mathrm{coil}}.$$

where $\sigma$ is the conductivity of the tissue, ${{\varvec{E}}}_{i}$ is the three dimensional electrical field of coil $i$, and the complex conjugate is denoted with a bar above the variable. All variables inside the integral are position dependent. The quantity ${\overline{{\varvec{x}}} }^{T}\phi {\varvec{x}}$ was used to calculate the power deposition matrix for a specific drive vector ${\varvec{x}}$.

## Results

**Fig. 3:**
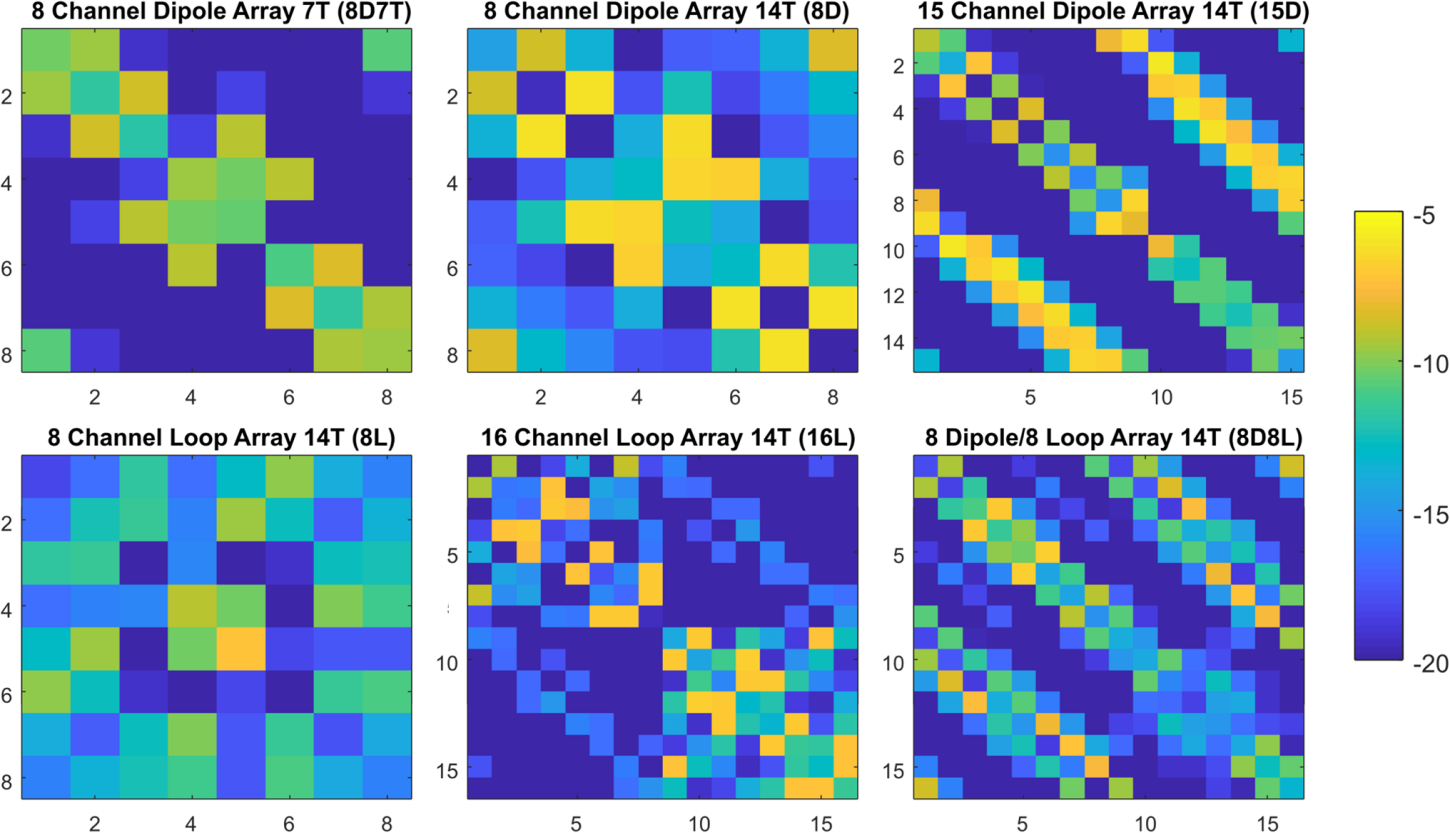
Scattering matrices of the proposed coil designs. At 14 T the coupling is more prominent for all arrays than at 7 T. All results have been obtained without additional decoupling mechanisms

The different coil designs were compared using the results of optimal RF shim drive vectors and the *k*_*T*_-points pulses. First the results from the RF shim solution are presented, followed by the results of the *k*_*T*_-points method. Furthermore, the scattering matrices are displayed in Fig. 3.

### RF shimming

**Fig. 4:**
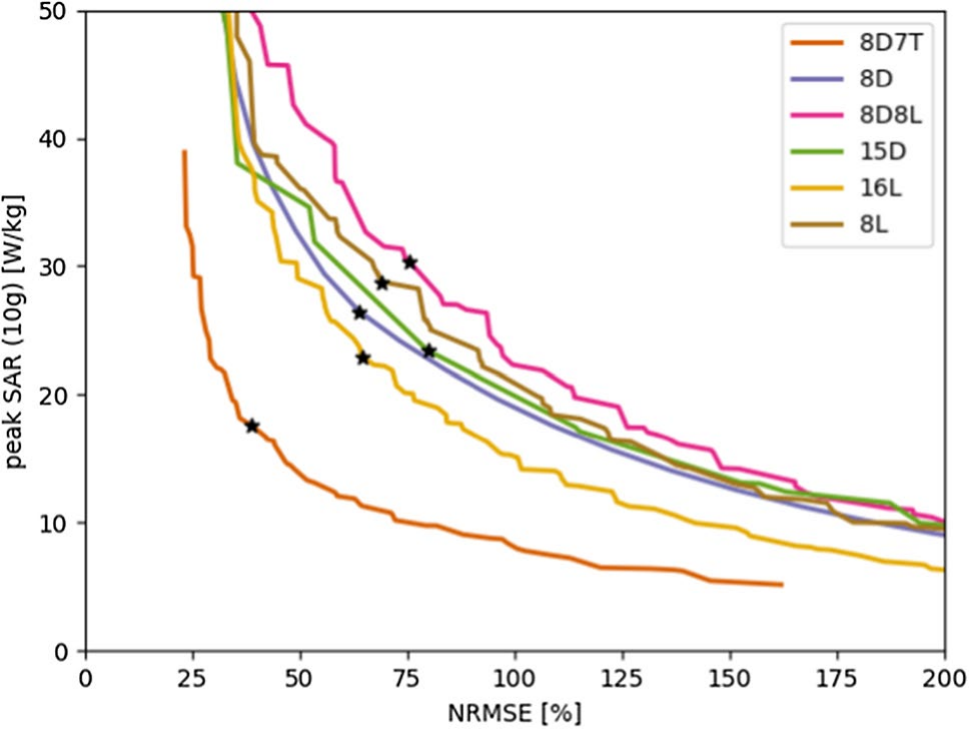
Result from the L-curve method when optimizing RF shim coefficients. The optimal regularization parameter is found at the point of strongest curvature, denoted by the black star. The corresponding RF shim is used for final evaluation

The L-curves of the optimization for RF shim coefficients are shown in Fig. 4 for each coil array. The peak SAR and NRMSE values are shown for all the 25 solutions. Based on this, the 16L array shows the best performance. However, in the vicinity of its optimal solution we do find that all other coil arrays are able to show similar performance.

**Fig. 5:**
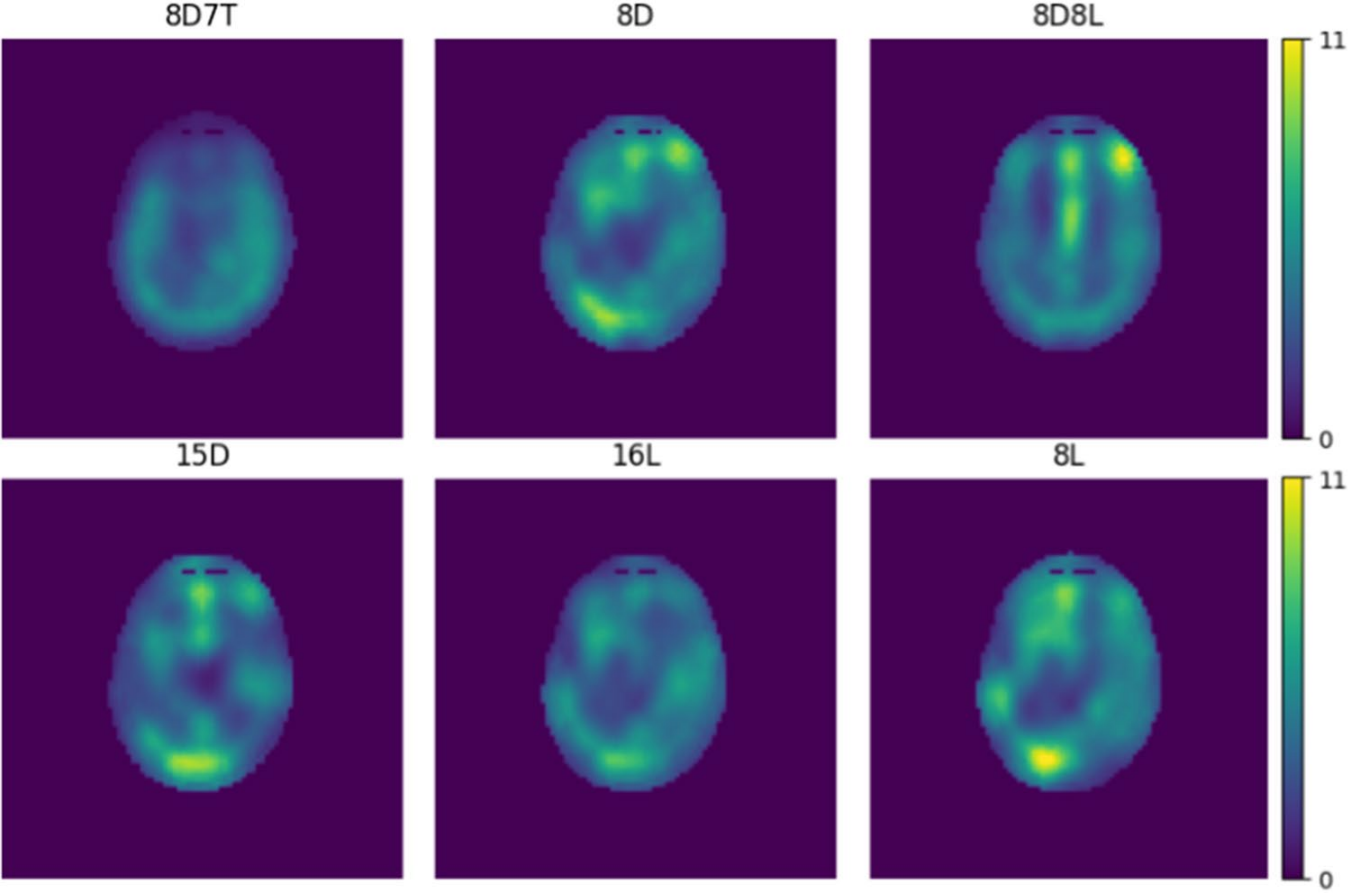
Using the optimal RF shim coefficients we evaluated the local SAR distributions, where we normalized to a head SAR of 3.2 W/kg

A visual impression of the SAR distribution for the selected optimal RF shims is given in Fig. 5 and a numerical comparison of the transmit performance for each coil design is given in Table 1.

**Table 1:** B_1_ performance metrics for the optimal RF shim drive vectors

	8 channel dipole array 7 T (8D7T)	8 channel dipole array (8D)	8 dipole 8 loop array (8D8L)	15 channel dipole array (15D)	16 channel loop array small (16L)	8 channel loop array big (8L)
Peak SAR [W/kg] (FA = 40°, D = 100%)	17.6	26.5	30.3	23.4	22.9	28.8
B1 + CoV [%]	27.4	33.8	30.0	32.0	33.5	36.5
Head SAR [W/kg] (FA = 40°, D = 100%)	7.8	8.2	8.7	7.3	8.3	7.9
Peak SAR [W/kg] (SAR_head_ = 3.2 W/kg)	7.2	10.3	11.1	10.2	8.8	11.7
Duty cycle [%] (SAR_head_ = 3.2 W/kg)	45	31	29	31	36	27

The shown metrics in this table have different normalizations, denoted by the round brackets. We normalized on a head SAR of 3.2 W/kg or on an B_1_^+^of 3.4µT

Table 1 shows that the peak SAR for a 40° flip angle at 14 T are approximately a factor 1.5–2 times higher than at 7 T, while minor differences are observed between the proposed coil designs. The level of homogeneity reached for all coil designs is also relatively similar.

Normalizing with respect to head SAR shows that the proposed designs at 14 T have a 20–40% increase in peak SAR compared to the 7 T array. We do observe that with this normalization all arrays are below the peak local SAR constraint of 10W/kg (normal operation mode) using a 100% duty-cycle with an average B_1_^+^ of 3.4 µT. In terms of maximum allowed duty cycle and head SAR levels we see that the 8D8L and 8L array have a reduced performance compared to the other designs.

**Fig. 6:**
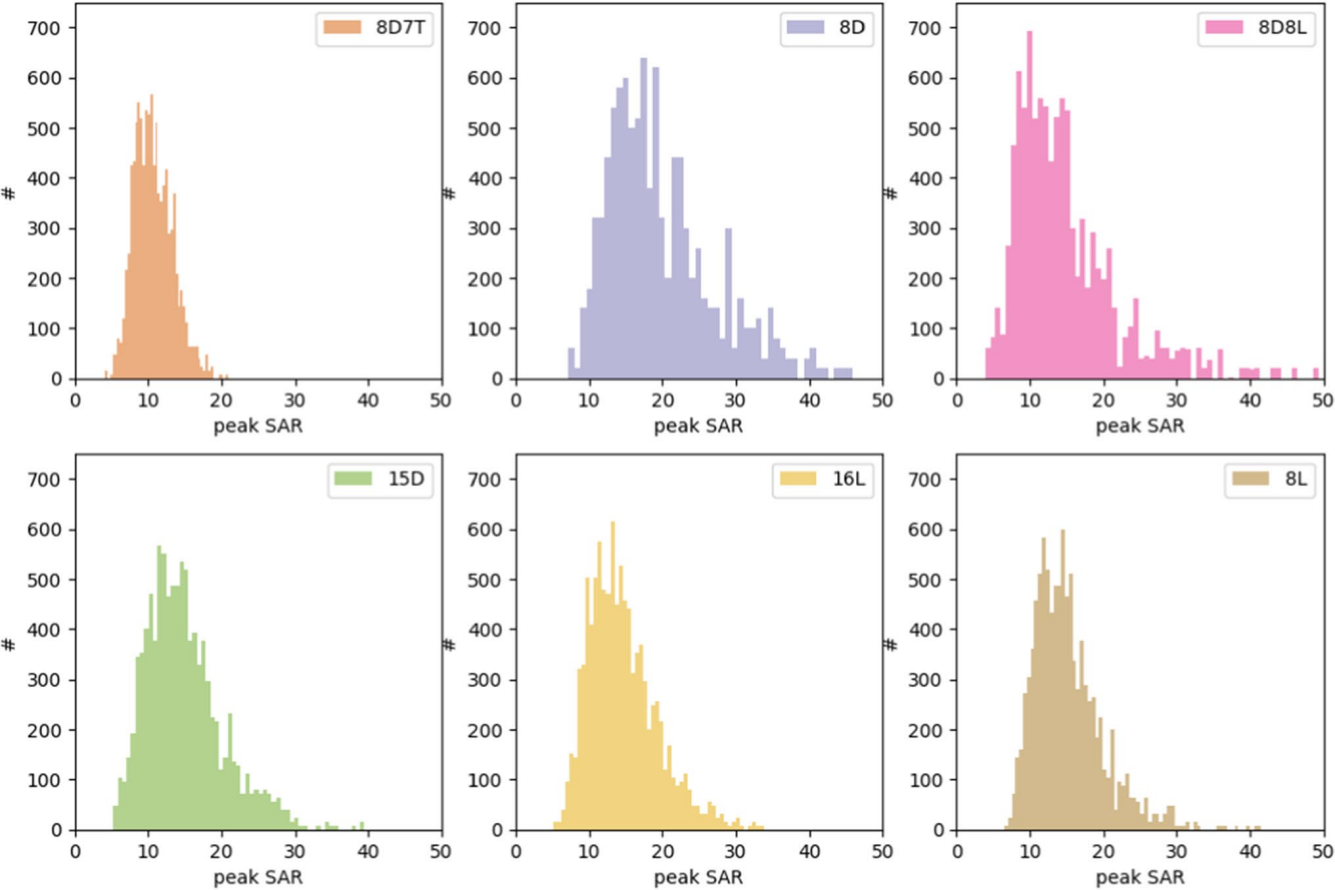
Peak SAR distribution per coil for a set of 10 000 random shims. For these shims a random phase and amplitude was chosen where the amplitude ranged between 0 and 4

The distribution of SAR for the 10 000 randomly generated shims is presented in Fig. 6. These show how the SAR at 14 T relates to 7 T, where we see an average increase of a factor of 1–2. The shape of the histogram shows that at 14 T the distribution has a longer tail towards the higher SAR values. Further, the difference in mean value is minimal for each coil design where we see a range of 15–20 W/kg.

### *k*_*T*_ -points

**Fig. 7:**
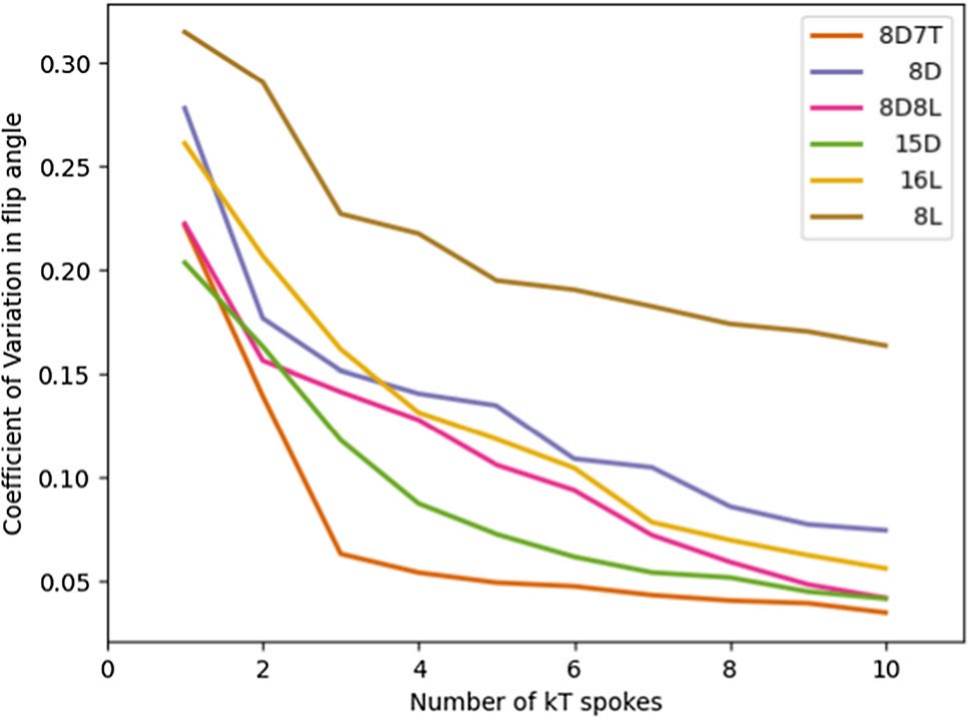
Coefficient of variation of the flip angle map for an increasing number of kT-points. Here the minimization problem was regularized with the forward power and the coefficient of variation is evaluated over the brain mask

The choice of the number of *k*_*T*_-points is derived from the results shown in Fig. 7. Here the coefficient of variation of the flip angle map is shown over the whole brain for each solution per coil design using a range of *k*_*T*_-points. This figure shows that pulses with more than 5 *k*_*T*_-points do not offer much improvement in homogeneity of the flip angles.

**Fig. 8:**
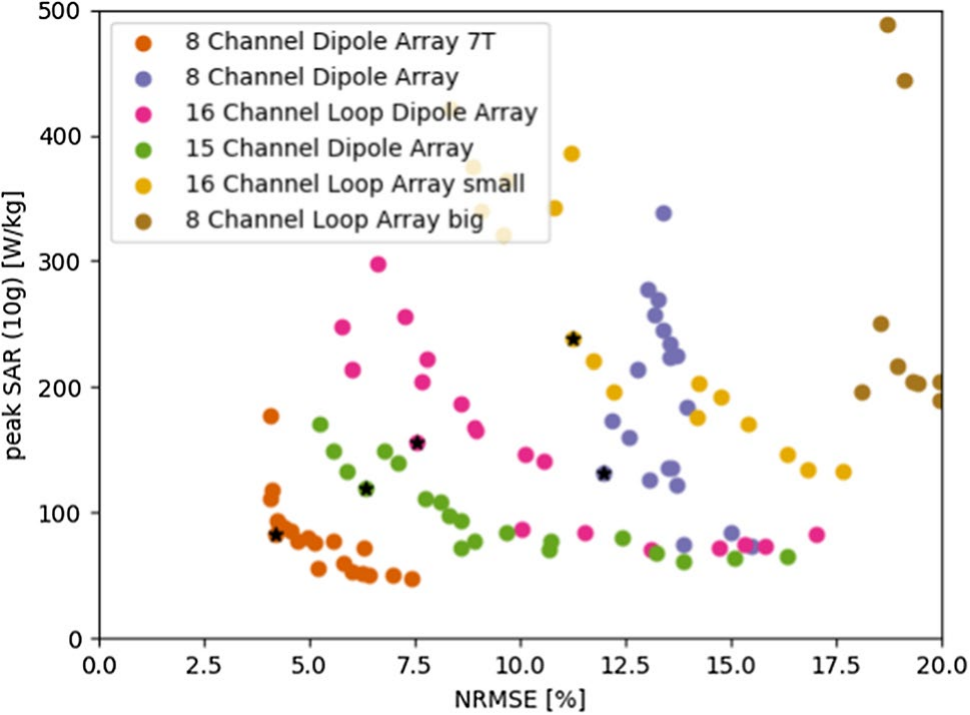
Result from the L-curve method when optimizing for 5 k_T_-points. Although the different solutions do not follow a smooth curve, the overall performance demonstrates an advantage for the dipole coil array designs compared to the loop coil array designs. The chosen optimal setting for each coil is denoted with a black star

In Fig. 8, we demonstrate the trade-off between the peak SAR and flip angle homogeneity. Although the resulting ‘curve’ suffers from discontinuities, the curves exhibit a particular order. Apart from the 7 T curve that is used for reference, we observe that coil arrays with 15 or 16 channels outperform all coil arrays with 8 channels, and that the dipole arrays show an improved performance compared to the loop coil arrays. This order of performance is also in line with the homogeneity found over the ranges of *k*_*T*_-points that is shown in Fig. 7.

**Fig. 9:**
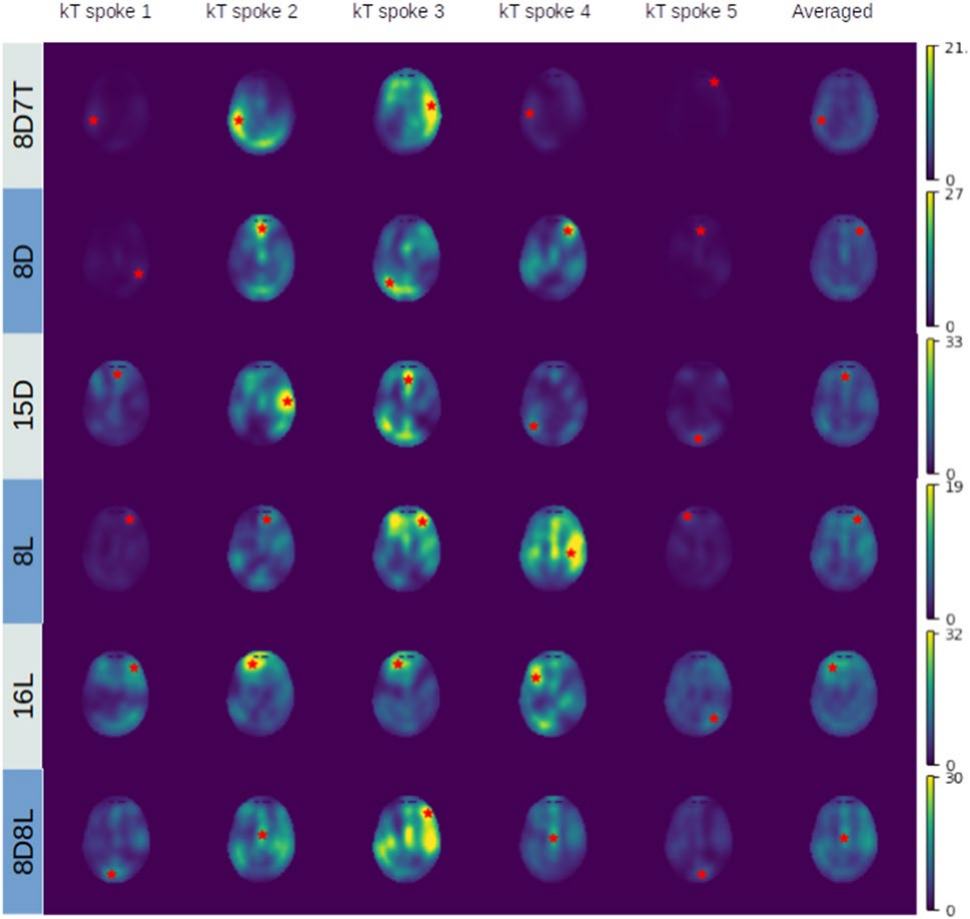
The SAR distribution of each individual point for the optimal 5-point k_T_ pulse normalized to 3.2 W/kg. The red star in each images shows the location of the peak SAR, notice how this changes location for each consecutive point

The different SAR distributions of each *k*_*T*_-points subpulses are displayed in Fig. 9. Notice how the peak SAR location changes across the 5 different subpulses, which reduces high peak SAR values in the time-averaged SAR distribution.

A numerical comparison between the proposed coil designs is given in Table 2. Notably, the *k*_*T*_-points pulse shows a strong improvement in the homogeneity of the flip angle compared to the optimal RF shim drive vectors. However, when using *k*_*T*_-points pulses, more power is required which is reflected by the increase in peak and head SAR levels.

**Table 2:** Numerical comparison of the 5 k_T_-points pulse analysis

	8 channel dipole array 7 T (8D7T)	8 channel dipole array (8D)	8 dipole 8 loop array (8D8L)	15 channel dipole array (15D)	16 channel loop array small (16L)	8 channel loop array big (8L)
Peak SAR [W/kg] (B_1_^+^ = 3.4 µT)	82.9	135.8	185.1	129.4	240.6	590.6
Flip angle CoV [%]	4.2	11.6	7.4	6.3	10.9	14.4
Head SAR [W/kg] (B_1_^+^ = 3.4 µT)	33.8	33.2	28.8	22.8	25.7	129.6
Peak sar [W/kg] (SAR_head_ = 3.2 W/kg)	7.8	13.1	20.6	18.2	30.0	14.6
Duty cycle [%] (SAR_head_ = 3.2 W/kg)	41	24	16	18	11	22

The shown metrics in this table have different normalization, denoted by the round brackets. We normalized on a head SAR of 3.2 W/kg or on an average B_1_^+^ of 3.4 µT

Further, we observe a stronger variance across the proposed coil designs. For example, the 8D8L and 15D array show one of the best homogeneity of the flip angle whereas the latter also has the lowest head SAR. On the other hand, the 8D has a relatively low peak SAR level and a beneficial duty cycle compared to the other proposed coil designs.

## Discussion

This work provides an exploratory view on the trade-off between SAR and flip angle uniformity for various coil designs at 14 T and a reference coil at 7 T. As expected, flip angle homogeneity, peak local SAR and head SAR all increase when moving from 7 to 14 T. Other findings, however, are less obvious.

Figures 4 and 8 show the trade-off between SAR and NRMSE for RF shimming and *k*_*T*_-points drive vectors. In both cases, it is clear that at 7 T, both lower NRSME and peak SAR values can be achieved. When RF shimming is used, the best performing 14 T array for lowest NRMSE is the 16L array. For RF shimming, differences between RF coils are small for the various coil arrays.

When using *k*_*T*_-points, the 15D array performs best, closely followed by the 8D8L array. Arrays with 15/16 transmit channels outperform the arrays with 8 channels and dipoles outperform loops coils. The latter result is in line with work at 7 T by van Leeuwen et al. [26], who demonstrated that dipoles reached lower peak SAR values than loop coils in unshielded head arrays. However, this pattern is less clear for RF shimming. The reason for this discrepancy is unclear. Possibly, because the dipole antennas have a larger penetration depth than the loop coils, they suffer from higher SAR levels because of stronger constructive interference of the electric fields. On the other hand, looking at Fig. 6, the 16L and 15D arrays have very much the same SAR histograms which contradicts this explanation.

Furthermore, the usage of various different drive vectors in *k*_*T*_-points averages out strong peaks in local SAR (Fig. 9), even though local SAR is not used as a regularization term when calculating the *k*_*T*_-points pulse. This finding highlights the impact of parallel transmission for 14 T brain imaging: using *k*_*T*_-points it becomes feasible to achieve uniform flip angles in the brain, while simultaneously alleviating local SAR hotspots. However, the use of *k*_*T*_-points also reduces overall power efficiency compared to normal RF shimming, therefore a lower duty cycle is allowed when using *k*_*T*_-points as compared to RF shimming.

Considering arbitrary shim settings, even when normalizing to a head SAR of 3.2 W/kg, peak local SAR on average becomes a factor of ~ 2.5-fold higher at 14 T than at 7 T, see Fig. 6. For 7 T, the peak local SAR limit is generally not exceeded when operating at the head SAR limit, for 14 T this is on average the case for 20% of all RF shims. However, when trying to achieve a uniform flip angle with either RF-shimming or *k*_*T*_-points, for most RF coils the global head SAR limit is exceeded before the local SAR limit, even at 14 T. Only when using *k*_*T*_-points and the 16L and the 8D8L array, the first level controlled mode peak SAR limit of 20 W/kg is exceeded at a head SAR of 3.2 W/kg (Table 2).

Compared to available literature, the simulation study in this manuscript has several limitations which need to be placed in perspective and can be addressed in future studies. First, the simulated coil arrays exhibit fairly strong inter-element coupling, especially the arrays with more than eight transmit channels. This likely leads to an underestimation of the RF shimming performance, especially for arrays with more than eight transmit channels. Although the loop coils were decoupled with nearest-neighbor decoupling, this did not reduce coupling between next-nearest neighbors or coils in the feet-head direction. Simulating the coils ideally decoupled [23, 32], would have likely introduced overestimation of the shimming degrees of freedom as full decoupling can never be achieved in a real coil array. Another option would have been the introduction of decoupling capacitors or inductors into the simulation, however, complex decoupling strategies will be required for next neighboring elements. Furthermore these decoupling strategies would apply mainly to the loop coils and are less applicable to arrays that include dipole antennas.

In general, it is assumed that because of stronger tissue loading, higher field strengths will lead to decreased inter-element coupling. In this work, we show that coupling does not strongly decrease at 14 T compared to 7 T and for some 14 T arrays could even increase compared to the reference 7 T array. Possible explanations include waveguide modes that propagate within the bore or increased reflection of the signals from the coil array at the air-tissue interface. Note that since we did not exhaustively investigate all possible array sizes and configurations, definite statements about coupling levels at various field strengths cannot be made.

Increased coupling may reduce the degrees of freedom when using RF shimming or pTx, which would then translate into suboptimal flip angle homogeneity or higher peak local SAR levels. Also, coupling impacts head SAR levels because scattered power is not deposited in the patient resulting in lower head SAR levels. However, coupling will also reduce B_1_ levels so the reduced head SAR with coupling is not likely to be advantageous. All these effects are included in the presented L-curves. In practice, if coupling deteriorates performance, the coil array may be built with decoupling circuitry. This has not been investigated in this study.

Another point that was not fully addressed in this study is the complete range of freedom in type, geometry and arrangement of the RF coil elements. For example, by modifying the loop coil elements into loopholes [33], self-decoupled coils [34] or coaxial loops [35–37], inter-element coupling could have been reduced. Dipole antennas can be decoupled by introducing passive decoupling elements [38], metamaterial structures [39] or using folded dipole antennas in combination with an RF shield [40]. Various methods have been suggested to reduce SAR for dipole antennas [3, 4, 39, 41, 42], this could further improve array performance. The length and width of the antennas considered in the current manuscript have been based on previous optimization studies [25, 26], however, these studies were not specifically aimed at 14 T brain imaging. A parametric study involving a more complete range of antenna lengths and width will likely show directions of further improvement. Different coil elements such as meander strip-line antennas [43], coaxial dipoles [44] or folded dipoles [41] could be another method to improve transmit performance. Finally, the coil arrays in this work include a 62 cm diameter RF shield (gradient shield) and are placed on a 30 cm diameter ring. Recent work by Zhang et al. [45] has indicated that for increasing field strength, the diameter of the RF shield has a very strong impact on SNR, and that the use of cylindrical RF transmit coils reduces the SNR of closely fitting receive arrays. It is likely that the implications of this work also hold for these transmit coil arrays.

In addition, improvements can be made in the methodology for solving the RF shimming coefficients. By solving for an absolute valued target vector the problem turns non-convex and increasingly difficult to solve for a global optimum. Either reformulating the problem to a convex one, by choosing a reasonable complex valued target B_1_ distribution or an algorithm that is better able to solve for non-convex minimization problems. In addition, there is room for improvement in the optimization of the RF shimming coefficients. Although solving the regularized magnitude least squared (MLS) problem has potential benefit [46], there are drawbacks to the current approach.

First, by regularizing only on forward power we are not able to conclude that an optimal peak SAR has been reached as well. Therefore, additional experiments are needed that regularize on peak SAR and show the trade-off between peak SAR and flip angle homogeneity.

Second, opposed to the current Tikhonov regularization, algorithms that enforce hard constraints can be beneficial to adhere to the SAR restrictions. For example, the work of [47] has used a primal–dual interior point method and stated that the results benefit from a constraint on both power and SAR restrictions. Moreover other work demonstrates that SAR reducing optimization strategies can improve performance [48, 49].

For sake of completeness, L-curves when regularizing on peak local SAR are depicted in supplementary Figs. S1 and S2. Note that in our work, the use of regularization on peak SAR levels makes the optimization landscape very irregular often resulting in solutions ending up in local minima and staggered L-curves. Therefore, smooth L-curves were created using a range of different starting values for each value of the Tikhonov parameter. The results show a reduced peak local SAR value for all coil arrays. However, the relative performance of all coil arrays stays more or less the same (Supplementary Fig. S1).

However it remains unclear whether such improved optimization strategies reveal a difference in the relative performance between the proposed coil designs.

As discussed above, there are still many design possibilities to explore for 14 T head coil arrays. With this work, we provide initial directions in choosing coil designs that improve flip angle homogeneity and reduce local SAR. Moreover, we demonstrate that at 14 T, the choice of RF coil has an impact on the achievable uniformity and peak SAR.

## Conclusion

We investigated the trade-off between SAR and flip angle uniformity for various head coil designs and parallel transmit strategies at 14 T in comparison to 7 T. We demonstrate that the type of coil element and the number of transmit channels has a strong impact on the transmit performance at 14 T. For arbitrary drive vectors, peak SAR is on average 2.5 times higher at 14 T than at 7 T. When operating at the head SAR limit of 3.2 W/kg, the peak SAR limit is exceeded for 20% of all drive vectors at 14 T, whereas at 7 T the peak SAR limit is almost never exceeded. However, when using either RF shimming or *k*_*T*_-points to achieve a uniform flip angle in the brain, head SAR and not peak SAR becomes the limiting factor for 14 T head imaging. Especially for *k*_*T*_-points pulses, high head SAR leads to a reduction of the maximum achievable duty cycle. Nonetheless, to achieve a uniform flip angle at 14 T, *k*_*T*_-points pulses are required. Using a 5-spoke *k*_*T*_-points results in a reduction of the flip angle coefficient of variation from 30 to 6% for the best performing array (15D) at 14 T, for a maximum duty cycle of respectively 31% and 18%. Consequently, a relatively high duty cycle of uniform flip angles can be obtained within SAR guidelines in the human brain at 14 T.

## Supplementary Information

Below is the link to the electronic supplementary material.

Supplementary Figure S1 A comparison between the L-curves resulting from regularizing on forward power and on peak SAR. The left figure shows all the found solutions for both methods, since the solver was not able to achieve the global minima several initial solutions were used which resulted in this scatter plot. The right figure shows the curve that runs along the minimum of all the solutions, creating a smooth L-curve which approximates the true global minimum solutions (PNG 351 KB)

Supplementary Figure S2 Result from the L-curve method when optimizing RF shim coefficients while regularizing on peak SAR (PNG 39 KB)

## Data Availability

The simulation data that was generated and analyzed during the current study are available from the corresponding author on reasonable request.
