# Supplementary figures and images for: Simulation-based evaluation of SAR and flip angle homogeneity for five transmit head arrays at 14 T
Source: MAGMA. 2023 Mar 31;36(2):245–55. doi: 10.1007/s10334-023-01067-1 (PMC10140109; doi:10.1007/s10334-023-01067-1)

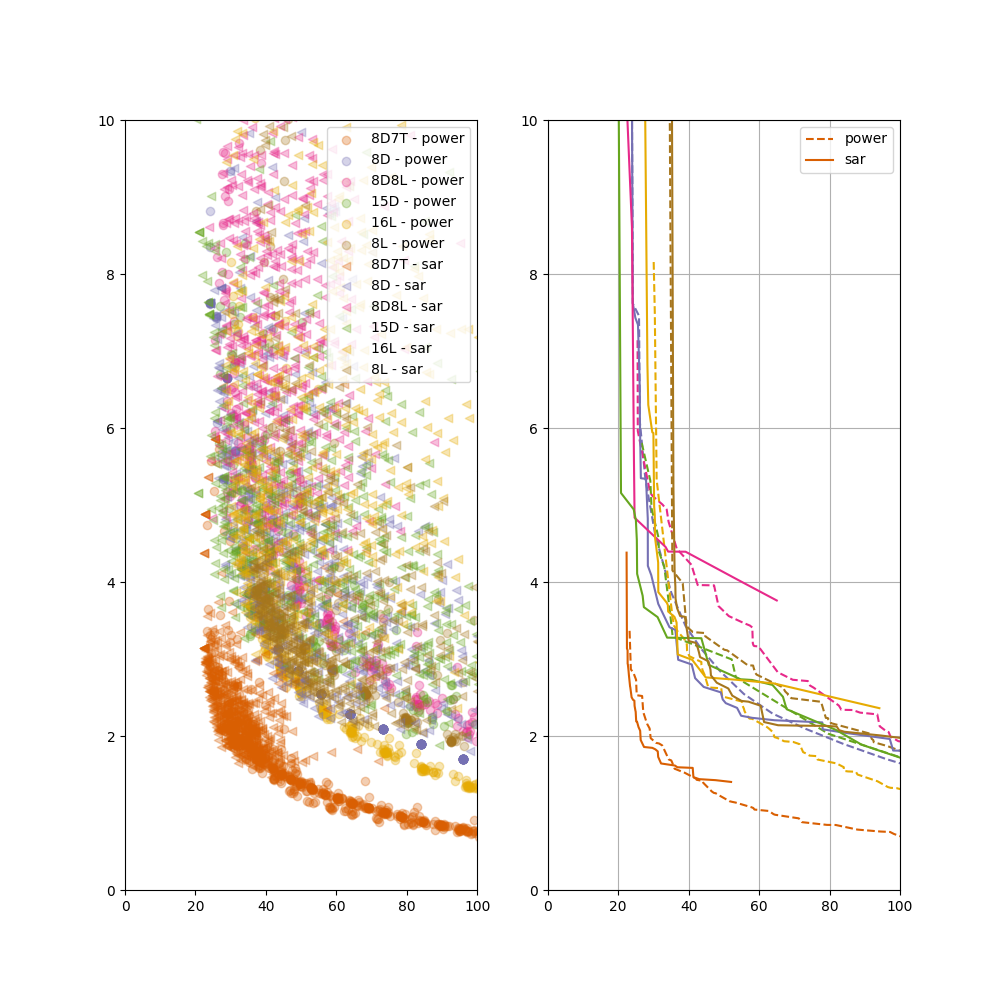

Supplement: Supplementary file 1 — Supplementary Figure S1 A comparison between the L-curves resulting from regularizing on forward power and on peak SAR. The left figure shows all the found solutions for both methods, since the solver was not able to achieve the global minima several initial solutions were used which resulted in this scatter plot. The right figure shows the curve that runs along the minimum of all the solutions, creating a smooth L-curve which approximates the true global minimum solutions (PNG 351 KB) [file 10334_2023_1067_MOESM1_ESM.png]

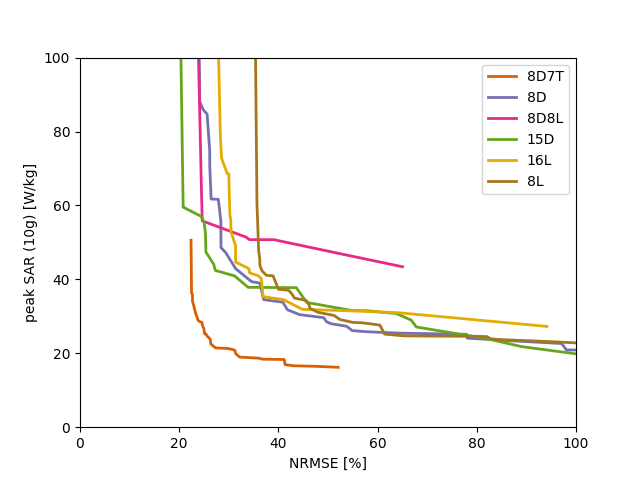

Supplement: Supplementary file 2 — Supplementary Figure S2 Result from the L-curve method when optimizing RF shim coefficients while regularizing on peak SAR (PNG 39 KB) [file 10334_2023_1067_MOESM2_ESM.png]
